# Direct NLRP3 inflammasome inhibitors for cardiovascular therapeutics: a comparative review of synthetic and natural candidates

**DOI:** 10.3389/fphar.2026.1861169

**Published:** 2026-06-24

**Authors:** Dong Zhen, Chunyan Liu, Xiao Sun, Terigen Bao, Bo Wang, Kai Zheng, Zhe Wu, Xiaojun Chen, Guoyou Zhang

**Affiliations:** 1 Tongliao People’s Hospital, Tongliao, Inner Mongolia, China; 2 Affiliated Tongliao Clinical Medical College of Inner Mongolia Medical University, Tongliao, Inner Mongolia, China

**Keywords:** cardiovascular diseases, direct inhibitors, drug discovery, medicinal plants, natural products, NLRP3 inflammasome

## Abstract

The NOD-like receptor family pyrin domain-containing 3 (NLRP3) inflammasome has emerged as a critical mediator in cardiovascular diseases, connecting cholesterol crystals and metabolic danger signals to atherogenic inflammation, myocardial injury, and adverse remodeling. Using direct inhibitors that bind to NLRP3 has advantages over indirect strategies. These advantages include retaining the function of other inflammatory body sensors and the potential for oral treatment. Several synthetic compounds have progressed to clinical trials. Recently, NT-0796 demonstrated a significant reduction in C-reactive protein in obese patients with cardiovascular risk, and VTX2735 has entered Phase II trials for recurrent pericarditis. However, the clinical failure of MCC950 due to hepatotoxicity underscores persistent translational challenges in this field. Oridonin is the most rigorously validated natural product that covalently modifies NLRP3 at Cys279. However, most reported “natural NLRP3 inhibitors” did not bind biochemically. This review demonstrates a three-tier evidence classification that differentiates confirmed direct inhibitors from functional modulators. We critically explored the potential for cardiovascular applications and identified bioavailability limitations, biomarker gaps, and trial design challenges that require innovative solutions.

## 1 Introduction

### 1.1 NLRP3 inflammasome

The NOD-like receptor family pyrin domain-containing 3 (NLRP3) inflammasome is a multiprotein cytosolic sensing platform that regulates inflammatory responses in cells due to endogenous or exogenous danger signals. It plays a key role in regulating innate immunity and inflammatory responses. The NLRP3 inflammasome is composed of three core components: NLRP3 itself (the sensor protein), the adaptor apoptosis-associated speck-like protein containing a CARD (ASC), and caspase-1 (the effector protease) (Swanson et al., 2019). The assembly of this complex follows a classical two-signal paradigm that has been extensively characterized over the past 2 decades. The cell is prepared for inflammasome activation by a priming signal, which is frequently induced by Toll-like receptor or cytokine receptor activation. This signal upregulates the NLRP3 and pro-interleukin (IL)-1β expression via the NF-κB pathway. Subsequently, various stimuli, including extracellular ATP, nigericin, and particulate or crystalline materials, can provide the second signal, which induces NLRP3 conformational changes, oligomerization, and ultimately inflammasome assembly (Kelley et al., 2019).

It is imperative to emphasize the fundamental distinction between physiological NLRP3 activation and pathological hyperactivation. Under homeostatic conditions, transient activation is a beneficial host defense mechanism that supports pathogen clearance and tissue repair. Conversely, sustained or dysregulated activation transformed this protective mechanism into a driver of disease pathogenesis. Activated caspase-1 cleaves gasdermin D (GSDMD) and oligomerizes its N-terminal fragments, forming plasma membrane pores. These pores facilitate the release of pro-inflammatory cytokines, including IL-1β and IL-18, and induce inflammatory cell death known as pyroptosis (Shi et al., 2017). This dual nature (protective in the short term and detrimental upon chronic activation) requires careful consideration of the timing and specific pathological context when targeting NLRP3 therapeutically.

**FIGURE 1:**
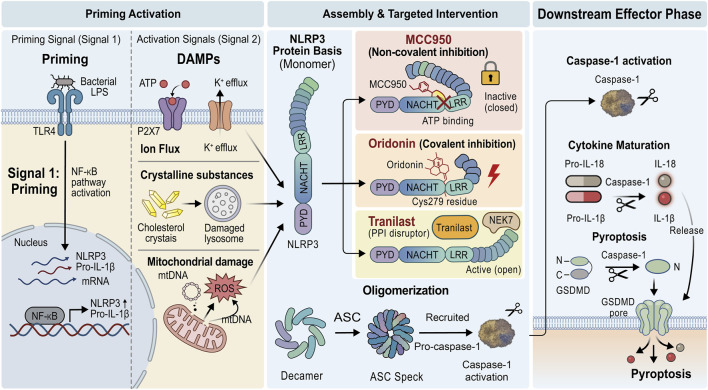
Activation pathway of the NLRP3 inflammasome and targeted mechanisms of direct inhibitors. Priming and Activation: The classical two-signal paradigm requires a priming signal (Signal 1), typically initiated by TLR4 activation, to upregulate NLRP3 and pro-IL-1β transcription via the NF-κB pathway. A subsequent activation signal (Signal 2) is triggered by diverse danger-associated molecular patterns (DAMPs), including K^+^ efflux, crystalline substances, and mitochondrial ROS or mtDNA. Assembly and Targeted Intervention: The NLRP3 monomer comprises PYD, NACHT, and LRR domains. Direct pharmacological inhibitors intercept assembly at specific structural checkpoints: MCC950 acts as a non-covalent inhibitor that locks the NACHT domain in an inactive, closed conformation; oridonin covalently modifies the Cys279 residue; and tranilast acts as a PPI disruptor to prevent NLRP3-NEK7 interaction. Downstream Effector Phase: Successful oligomerization leads to ASC recruitment and caspase-1 activation. This effector protease subsequently cleaves pro-IL-1β and pro-IL-18 into their mature forms and cleaves gasdermin D (GSDMD) to execute pyroptosis via membrane pore formation (Created with BioRender.com. Mechanism partially adapted from Toldo and Abbate, 2018).

To therapeutically target NLRP3, it is important to understand how it differs from the other inflammasome sensors within the broader nucleotide-binding domain and leucine-rich repeat-containing (NLR) superfamily. The NLR superfamily encompasses several members, including NLRP1, NLRP3, NLRC4, and non-NLR sensors like AIM2. A critical distinction lies in their activation specificity. For instance, NLRP1 is typically activated by specific bacterial toxins, AIM2 detects cytosolic DNA, while NLRC4 responds to bacterial components, such as flagellin (Chou et al., 2023; Sundaram et al., 2024). These sensors possess relatively well-defined, singular ligands and primarily mediate classical antimicrobial defenses. Conversely, NLRP is activated by a significantly diverse set of danger signals (Broz and Dixit, 2016). This diversity renders mechanistic studies more challenging, however, it also explains why NLRP3 is central to sterile inflammatory diseases, such as atherosclerosis and heart failure, as multiple metabolic and biomechanical disturbances converge on the same pathway (Figure 1). This multifaceted activation sequence underscores the necessity of identifying specific structural “checkpoints” where the process can be halted. Figure 1 provides an integrated perspective, mapping the transition from cellular signaling to the precise molecular pockets within the NLRP3 architecture—such as the NACHT domain and the NEK7 interface—that serve as the primary targets for direct pharmacological intervention. By visualizing these binding sites alongside the assembly process, we establish a structural framework for understanding how small molecules physically intercept the inflammatory cascade. Conversely, selective inhibition of NLRP3 may alleviate this pathological inflammation while preserving other inflammasome sensors (such as AIM2 and NLRC4), though it must be noted that prolonged NLRP3 suppression still carries specific opportunistic infection risks and provides relative, rather than absolute, safety.

### 1.2 NLRP3 and cardiovascular disease: From association to causation

Although we have a comprehensive biological understanding of the function of NLRP3 in human diseases, its crucial role in cardiovascular disease has been supported by numerous lines of evidence from mechanistic studies, genetic models, and clinical observations. The seminal study by Duewell identified cholesterol crystals as potent activators of NLRP3 (Duewell et al., 2010). This finding primarily repositioned the NLRP3 inflammasome from only a component of antibacterial defense to a central mediator of atherogenic inflammation. Furthermore, it provided a molecular explanation for the long-observed inflammatory component of atherosclerosis, whose mechanism was previously unknown. Subsequent studies confirmed the persistent activation of NLRP3 in macrophage-derived foam cells in the plaque (Duewell et al., 2010; Yalcinkaya and Tall, 2025). This process triggered the IL-1β-induced inflammatory cascade and promoted endothelial dysfunction, smooth muscle cell migration, and extracellular matrix degradation, key pathological processes in plaque progression and instability. These findings are also corroborated by human pathological studies, demonstrating significantly higher NLRP3 expression in vulnerable plaques than in stable plaques. The inflammasome components are concentrated in the rupture-prone lipid-rich necrotic core and shoulder regions (Paramel Varghese et al., 2016). The clinical relevance of this pathway was critically validated by the momentous CANTOS trial, which demonstrated that IL-1β neutralization with canakinumab reduced recurrent cardiovascular events in patients after myocardial infarction (MI), despite the high cost and increased infection-associated mortality, which reduced its initial treatment enthusiasm (Ridker et al., 2017).

In addition to chronic atherosclerosis, NLRP3 activation is crucial in the progression of acute coronary syndromes, particularly during myocardial ischemia-reperfusion injury. During acute MI (AMI) and reperfusion, damaged cells release a group of DAMPs, including extracellular ATP, high-mobility group box 1, and mitochondrial DNA. These DAMPs collectively induce robust NLRP3 activation in surviving cardiomyocytes and infiltrating immune cells (Toldo and Abbate, 2018). Cardiomyocyte pyroptosis in the infarct border zone initiates a self-amplifying “bystander effect,” in which dying cells release additional DAMPs, thereby expanding the injury beyond the initial ischemic area. This feedforward mechanism significantly influences the final infarct size and subsequent adverse cardiac remodeling, suggesting that NLRP3 inhibition may have a protective effect during this time window.

In heart failure, persistent low-grade inflammation, partially mediated by NLRP3, drives the myocardial fibrosis progression and poor structural remodeling over months to years. New evidence suggests that NLRP3 has cell-specific effects in the myocardium. Inflammasome activation promotes hypertrophy and systolic dysfunction in cardiomyocytes while accelerating collagen deposition and diastolic impairment in cardiac fibroblasts (Sano et al., 2018). These observations have significant therapeutic implications, as the benefit-risk profile of intervention may be optimized by targeting inflammasome activity in specific cell populations. Recent studies have further implicated NLRP3 in diabetic cardiomyopathy, where hyperglycemia and lipotoxicity converge to activate the inflammasome, and in atrial fibrillation (AF), where inflammation promotes pro-arrhythmic structural and electrical remodeling (Yao et al., 2018). These novel research areas require further investigation and represent potential avenues for future therapeutic development.

Despite this convincing evidence, a critical limitation was that most mechanistic evidence derives from genetic knockout or pharmacological inhibition in rodent models, with uncertain translatability to human disease. Although the CANTOS experiment significantly strengthens causal evidence in humans, the evidence for direct NLRP3 inhibition compared with downstream IL-1β blockade is inadequate. Mendelian randomization studies of NLRP3 pathway variants and genome-wide association studies of inflammasome gene loci in large cardiovascular cohorts are crucial to establish human relevance; however, they remain underexplored (Libby and Everett, 2019; Chen et al., 2023). Until such genetic evidence accumulates, caution is warranted regarding the precise benefit of direct NLRP3 targeting in human cardiovascular disease.

### 1.3 Evolution of NLRP3 inhibition strategies and the theoretical appeal of direct targeting

Multiple strategies target the NLRP3 inflammasome pathway and can be broadly categorized into indirect and direct inhibition. The selection of a strategy should be evaluated in the specific disease context.

Indirect inhibition strategies target upstream signaling events or downstream effector molecules but come with significant limitations. For instance, inhibition of upstream pathways, such as TLR4 or NF-κB, significantly suppresses innate immunity and increases the risk of infection. Interventions targeting common activation events, such as potassium efflux, may cause off-target effects due to a lack of specificity for NLRP3. The failure of large-scale clinical trials (for instance, with vitamin E and beta-carotene) demonstrates that “broad-spectrum” strategies targeting mechanisms, such as oxidative stress, have limited efficacy in cardiovascular diseases, highlighting the importance of target specificity (Sesso et al., 2008). Although clinically validated, monoclonal antibodies (such as canakinumab) that target the downstream effector IL-1β are expensive, require long-term monitoring, and have irreversible effects.

Conversely, the direct inhibition strategy uses small molecules that specifically bind the NLRP3 protein to block its activation. Theoretically, this method offers distinct advantages: it may selectively inhibit NLRP3 without affecting other inflammasomes, such as AIM2 and NLRC4, thereby suppressing pathological inflammation while maintaining the essential host defense against infections. Furthermore, small-molecule inhibitors often offer oral administration, flexible dosing, and manageable discontinuation in case of adverse effects.

However, the direct inhibition strategy also faces challenges and uncertainties. First, NLRP3 plays physiological roles in host defense and tissue repair, implying potential risks associated with its long-term inhibition (Mangan et al., 2018). Second, preclinical data must be interpreted cautiously, as even compounds with high *in vitro* selectivity (for instance, MCC950) can fail in clinical trials because of off-target effects or metabolic toxicity. Therefore, the ultimate efficacy and safety of direct NLRP3 inhibitors must be validated through rigorous clinical trials. However, it is important to emphasize that, despite the theoretical benefits of the direct NLRP3 inhibition in terms of target specificity, its superior efficacy or safety compared to indirect strategies (for instance, IL-1β blockade with canakinumab) remains unproven in humans. This distinction must be addressed in future head-to-head or biomarker-stratified trials.

### 1.4 Scope, review methodology, and classification criteria

The scope of this review is strictly focused on the pharmacological evaluation and cardiovascular translation of direct NLRP3 inflammasome inhibitors. This review particularly emphasizes direct NLRP3 inhibitors that physically bind the NLRP3 protein, distinguishing them from agents that act through indirect mechanisms, and provides clear mechanistic insights for therapeutic development. To ensure a comprehensive and unbiased evaluation, we conducted a systematic literature search across PubMed, Scopus, and ClinicalTrials.gov databases for articles published up to March 2026. The search strategy utilized combinations of keywords including “NLRP3 inflammasome,” “direct inhibitor,” “natural products,” “small-molecule,” and “cardiovascular disease.” The inclusion criteria focused on: (i) compounds with reported direct binding to NLRP3 or its essential co-factors (e.g., NEK7); (ii) studies providing clear structural or biochemical evidence of target engagement; and (iii) candidates evaluated in cardiovascular preclinical models or clinical trials. Studies focusing solely on transcriptional priming (Signal 1) without addressing the activation phase (Signal 2) were excluded.

Given the variable quality of evidence in the field, particularly for natural products, we use a three-tier classification system (“Confirmed,” “Probable,” and “Functional”) to rigorously evaluate candidates based on biochemical, structural, and functional data. The rules for assigning compounds to these tiers are defined as follows:

Tier 1 (Confirmed Direct Inhibitors): Requires definitive biophysical evidence of direct binding, such as X-ray co-crystallography, Cryo-EM structures, mass spectrometry-confirmed covalent modification (e.g., Cys279), or Surface Plasmon Resonance (SPR) data with purified NLRP3 protein.

Tier 2 (Probable Direct Inhibitors): Includes compounds demonstrating high-affinity binding in molecular docking simulations and significant target engagement in cellular assays (e.g., Cellular Thermal Shift Assay, CETSA), but lacking direct biophysical validation with purified proteins.

Tier 3 (Functional Inhibitors): Encompasses agents that suppress NLRP3 activation in phenotypic assays (e.g., reduced IL-1β release) but likely act through upstream signaling modulation (e.g., ROS scavenging, AMPK activation) or exhibit “promiscuous” multi-target activity without evidence of specific NLRP3 pocket binding.

By establishing synthetic inhibitors as mechanistic anchors under these standardized criteria, we provide a robust context to evaluate the therapeutic potential and structural optimization opportunities of natural products. Our distinct perspective involves systematically comparing structure-activity relationships between synthetic and natural inhibitors and using this mechanistic hierarchy grading framework to differentiate between established mechanisms and speculation, thereby informing rational compound prioritization.

## 2 Mechanisms, clinical progress, and lessons from synthetic small-molecule direct NLRP3 inhibitors

### 2.1 NLRP3 protein architecture and druggable binding sites

Recently, advances in cryo-electron microscopy (cryo-EM) and X-ray crystallography enabled the precise understanding of the three-dimensional structure of NLRP3 and its inhibitor complexes, which is required for the rational design of these products. The NLRP3 protein is composed of three linearly arranged domains: the N-terminal pyrin domain (PYD), which primarily mediates binding to the adaptor protein ASC; the central NACHT domain, a core catalytic unit that hydrolyzes ATP and drives inflammasome assembly; and the C-terminal leucine-rich repeat (LRR) domain, which is involved in maintaining autoinhibition and sensing danger signals (Sharif et al., 2019). The distinct functions and targetability of these three domains determine different strategies for inhibitor development.

The NACHT domain has emerged as the primary target for small-molecule inhibitors because of its well-defined ATP-binding and hydrolysis pockets. The transition of NLRP3 from an autoinhibited “closed” conformation to an activated “open” state is dependent on the coordination of ATP binding and hydrolysis through Walker A and B motifs within this domain (Duncan et al., 2007). MCC950 and other successful inhibitors act by binding to a pocket adjacent to the Walker B motif, thereby directly preventing the conformational rearrangements and oligomerization required for NLRP3 activation. In 2022, high-resolution cryo-EM structures of the NLRP3 decamer in complex with the inhibitor CRID3 (such as MCC950) were solved, establishing a crucial template for structure-based drug design (Hochheiser et al., 2022). However, the highly hydrophobic nature of this binding pocket poses a challenge, as it favors lipophilic lead compounds but often results in poor aqueous solubility and low bioavailability for drug candidates.

Targeting the PYD domain is more challenging because it regulates interactions with ASC through extensive protein-protein interfaces that are difficult to disrupt with small molecules. However, novel techniques, such as fragment-based drug discovery, offer potential strategies. In the autoinhibited state, the LRR domain folds back over the NACHT domain, suggesting that compounds that stabilize this conformation may provide an alternative, non-competitive inhibition mechanism. Furthermore, the interface between NLRP3 and its essential co-factor NEK7, involving both the NACHT and LRR domains, is another valuable target, as exploited by compounds such as tranilast (Huang et al., 2018).

### 2.2 Molecular mechanisms of established inhibitors

#### 2.2.1 MCC950: achievements and lessons from a pioneering inhibitor

MCC950 (also known as CP-456,773 or CRID3) was the first NLRP3 inhibitor to be extensively studied and highly selective. This diarylsulfonylurea-based small molecule demonstrates high target specificity, with nanomolar potency against NLRP3 *in vitro* and no activity against other inflammasomes, such as AIM2 and NLRC4 (Coll et al., 2015; Coll et al., 2019). Accordingly, it has become a key tool for studying NLRP3 function and has served as a lead structure for further development. However, its direct binding to endogenous NLRP3 was not experimentally confirmed until 2024, when Zhao et al. used chemical proteomics to demonstrate this interaction in human macrophages (Zhao et al., 2024). Mechanistically, MCC950 binds to the NACHT domain of NLRP3, located adjacent to the critical Walker B motif (Grant et al., 2023; Xu et al., 2023). It stabilizes NLRP3 in an inactive “closed” conformation, inhibiting its ATP hydrolysis activity and oligomerization, and interfering with the interaction between NLRP3 and its essential co-factor NEK7 (Tapia-Abellán et al., 2019). Notably, MCC950 selectively targets the inflammasome activation signal without affecting its transcriptional priming signal. MCC950 rapidly became the standard research tool for exploring NLRP3 function (>3,000 citations) and the lead scaffold for subsequent inhibitor programs.

MCC950 has demonstrated broad therapeutic potential in cardiovascular disease models. In atherosclerosis studies, it revealed a direct anti-inflammatory mechanism, significantly reducing plaque burden by approximately 50% and decreasing macrophage infiltration and IL-1β/IL-18 levels within lesions, without affecting lipid profiles (Duewell et al., 2010). MCC950 primarily suppresses macrophage pyroptosis by reducing GSDMD cleavage and pore formation in foam cells (Cabral et al., 2025). Furthermore, a recent study demonstrated that MCC950 reversed endothelial NLRP3 activation induced by lncRNA MIR181A1HG, extending its anti-atherogenic effects beyond macrophages (Ni et al., 2025).

In porcine myocardial infarction (MI) models, MCC950, administered before reperfusion or up to 6 h post-ischemia, reduced infarct size by 30%–40% and preserved left ventricular function (van Hout et al., 2017). In the infarcted area of mice, MCC950 reduced neutrophil infiltration, cardiomyocyte pyroptosis, and fibroblast activation (Toldo et al., 2019). Furthermore, it reduced the incidence rate of aortic aneurysm in an Ang II-induced model from 70% to 30% (Ren et al., 2020) and improved cardiac function in an heart failure with preserved ejection fraction (HEpEF) model, reducing hypertrophy by 19.5% and fibrosis by 32.5% (Li et al., 2024).

Despite these robust preclinical results, MCC950 failed clinical translation. In Phase II trials for rheumatoid arthritis, patients receiving high doses (1,200 mg daily) experienced hepatotoxicity, as evident by ALT/AST levels exceeding the upper normal limit three times, leading to trial termination (Mangan et al., 2018). The furan ring in MCC950 potentially produces reactive metabolic intermediates via CYP450, and the high-dose requirement exacerbates this liability. Furthermore, chemical proteomics revealed that MCC950 binds carbonic anhydrase 2 (CA2) with Ki values of 1–5 μM, a previously unrecognized off-target interaction that may cause adverse effects (Kennedy et al., 2021). Clinical experience has also highlighted the development challenges, such as high-dose requirements, solubility limitations, and unpredicted off-target effects, that continue to guide the field.

#### 2.2.2 CY-09 and NT-Series compounds: second-generation NACHT binders

The clinical failure of MCC950 directed medicinal chemistry efforts to develop improved NLRP3 inhibitors. CY-09, identified through virtual screening, is a direct ATP-competitive inhibitor (IC50 approximately 6 μM) that binds the NACHT nucleotide-binding pocket in a manner that partially overlaps with MCC950. It demonstrated efficacy in preclinical models of type 2 diabetes and gout (Jiang et al., 2017).

A distinct optimization strategy was used to mitigate a key limitation of MCC950: lack of central nervous system (CNS) penetration. NT-0796 is an isopropyl ester prodrug that is hydrolyzed by carboxylesterase-1 intracellularly, releasing the active metabolite NDT-19795 for NLRP3 inhibition in the brain (Harrison et al., 2023). Structure–activity work on these scaffolds confirmed that the sulfonylurea moiety is essential for NACHT binding, whereas modification of the terminal aryl group can improve selectivity over CA2. Unlike CY-09, which remains in preclinical stages, NT-0796 has successfully advanced to clinical evaluation. Recent Phase I/II trial data demonstrated its ability to penetrate the blood-brain barrier and significantly reduce peripheral inflammatory biomarkers, including C-reactive protein, in obese subjects with cardiovascular risk factors, marking a critical step forward for this scaffold. A persistent challenge that has not been satisfactorily resolved is to balance sufficient lipophilicity for NACHT pocket binding with adequate aqueous solubility for oral dosing.

### 2.3 Clinical-stage compounds and cardiovascular application prospects

Several NLRP3 inhibitors have entered clinical development. However, most trials have focused on non-cardiovascular inflammatory diseases, and the efficacy and safety of these agents in core cardiovascular indications, such as atherosclerosis and heart failure, remain to be established.

DFV890, developed by Novartis, is one of the more advanced candidates and has completed a Phase II trial in cryopyrin-associated periodic syndromes (Coll et al., 2022). Its mechanism of action is comparable to that of MCC950; however, its structure has been optimized to improve its drug-like properties. Currently, its potential for cardiovascular applications is primarily inferred from its mechanism, due to the lack of dedicated clinical trial data. The current development strategy of the company prioritizes rare diseases with smaller patient populations to accelerate regulatory approval.

Emlenoflast was developed by Inflazome (later acquired by Roche). Owing to its excellent blood-brain barrier penetration, primary research has focused on neurological diseases, including Parkinson’s disease (Roche, 2025). Although this may seem distant from cardiology, the drug may have potential applications at the intersection of cerebrovascular and cardiac diseases, given its role in inflammation during post-stroke recovery and in vascular cognitive impairment. Although preliminary clinical trials have demonstrated an acceptable safety profile, there is a dearth of long-term tolerability data.

Dapansutrile is the most relevant to the cardiovascular field. A developing company, Olatec Therapeutics, has conducted early-phase (Phase Ib/IIa) clinical trial in patients with systolic heart failure and elevated inflammatory biomarkers (Yang et al., 2019). Its rationale for its application is to improve cardiac remodeling by suppressing persistent myocardial inflammation. Good tolerability in preliminary data presents an opportunity for larger efficacy studies. Contrary to the orphan disease strategy adopted by many large pharmaceutical companies, Olatec is directly targeting the high-unmet-need area of heart failure, which entails greater developmental risks but also addresses a major clinical burden.

Tranilast presents a unique case. It is an anti-allergic drug that has been marketed in Japan for decades. Its ability to inhibit the NLRP3-NEK7 interaction was later discovered (Huang et al., 2018). It has been used in Japanese clinical practice to prevent coronary stent restenosis and has amassed extensive long-term safety data that are unparalleled by any novel inhibitor. However, its potency against NLRP3 is relatively modest, and its clinical benefits from NLRP3 inhibition versus other mechanisms remain unclear. Therefore, it is challenging to interpret its cardiovascular benefits as definitive proof of concept for NLRP3-targeted therapy.

VTX2735, under development by Ventyx Biosciences, is the first NLRP3 inhibitor directly targeting a cardiovascular inflammatory condition that has recently entered Phase II trials for recurrent pericarditis (Ventyx Biosciences, 2025). Although pericarditis is less prevalent than atherosclerotic disease, it offers advantages as an initial cardiovascular indication. The condition is often recurrent and highly symptomatic, enabling a relatively rapid assessment of therapeutic effects. Furthermore, current therapeutic options are limited following the failure of standard anti-inflammatory approaches. Although the trial is currently active, definitive data regarding its ability to prevent clinical flares and reduce corticosteroid dependence are still pending. Successful outcomes in this indication could provide the clinical validation required to justify larger trials in more prevalent cardiovascular diseases.

Selnoflast (also known as RO7486967), an orally bioavailable NLRP3 inhibitor developed by Roche, represents another potent NACHT domain binder entering the cardiovascular clinical pipeline. Specifically, selnoflast is being investigated for its capacity to mitigate residual inflammatory risk in patients following acute myocardial infarction (AMI) —a critical clinical window where pathological NLRP3 hyperactivation drives adverse ventricular remodeling. A recent Phase II trial evaluating selnoflast in post-MI patients with elevated residual inflammation (hsCRP ≥2 mg/L) demonstrated encouraging target engagement, revealing a 36% reduction in circulating IL-6 and a 70% reduction in hsCRP levels without significant safety concerns (Kunder et al., 2025). However, like other contemporary synthetic candidates, its definitive efficacy in reducing hard cardiovascular events remains to be established in appropriately powered, long-term outcome trials.

Table 1 summarizes the current landscape of clinical-stage NLRP3 inhibitors, including their mechanisms, developmental status, and cardiovascular relevance.

**TABLE 1:** Clinical stage direct NLRP3 inhibitors: Development status and cardiovascular relevance.

Compound	Developer	Clinical stage	Primary indication	Mechanism	CV relevance	Key limitations
MCC950	Pfizer/IFM	Terminated (phase II)	Rheumatoid arthritis	Direct NLRP3 inhibitor (NACHT binding)	High	Hepatotoxicity; CA2 off-target
DFV890	Novartis	Phase II	CAPS; myeloid diseases	Direct NLRP3 inhibitor (NACHT binding)	Moderate	No dedicated CV trials
NT-0796	NodThera	Phase I/II	Obesity with CV risk	Direct NLRP3 inhibitor (NACHT binding)	High	CRP reduction demonstrated
Emlenoflast	Roche	Phase II	Parkinson’s disease	Direct NLRP3 inhibitor (NACHT binding)	Low-moderate	CNS focus
Dapansutrile	Olatec	Phase Ib/IIa	Heart failure; gout	Direct NLRP3 inhibitor	High	Efficacy data pending
VTX2735	Ventyx	Phase II	Recurrent pericarditis	Direct NLRP3 inhibitor	High	Early stage
Selnoflast	Roche/Inflazome	Phase II	Post-MI; atherosclerosis	Direct NLRP3 inhibitor (NACHT binding)	High	Hard outcome (MACE) data pending
Tranilast	Kissei	Marketed (Japan)	Allergic disorders	NLRP3-NEK7 interaction modulator	High	Modest potency (IC50 20–50 μM)

Abbreviations: CAPS, cryopyrin-associated periodic syndromes; CV, cardiovascular; BBB, blood-brain barrier; CA2, carbonic anhydrase 2.

### 2.4 Critical obstacles in clinical development

Although NLRP3 inhibitors have robust preclinical evidence, their clinical translation is slow, reflecting several fundamental challenges.

The unpredictable nature of off-target toxicity remains a key challenge. MCC950 exhibited exceptional selectivity in standard screens, with IC50 ratios exceeding 1000-fold against other inflammasomes and no significant broad-panel receptor-binding activity. However, Phase II trials revealed dose-limiting hepatotoxicity, unexpected by preclinical safety studies in mice, rats, and dogs (Muela-Zarzuela et al., 2024). This species-specific toxicity is potentially due to differences in hepatic metabolism but illustrates the limited predictive power of current preclinical models. Subsequently, chemical proteomics demonstrated that MCC950 binds CA2 at therapeutic concentrations, potentially contributing to adverse effects via inflammasome-independent mechanisms (Kennedy et al., 2021). This highlights the importance of early target deconvolution using approaches such as cellular thermal shift assays and affinity proteomics to identify off-target interactions before clinical trials.

Biomarker validation gaps significantly complicate trial design, particularly for cardiovascular applications that require years of endpoint follow-up. Contrary to low-density lipoprotein-cholesterol in lipid-lowering therapy, there is no equivalently validated biomarker for NLRP3 inhibition. Although plasma IL-1β levels are mechanistically relevant, their use as response markers is limited due to high intra-individual variability. High-sensitivity C-reactive protein, validated in the CANTOS, reflects general inflammation rather than specific NLRP3 activity and is unable to differentiate between on-target and off-target effects (Ridker, 2016). Novel candidates, such as circulating GSDMD fragments, percentages of NLRP3-positive monocytes, and exosomal inflammasome components, remain under investigation and lack the prospective validation necessary for regulatory approval.

In addition to drug-drug interactions, integrating established therapies poses practical challenges. Patients routinely receive statins, antiplatelet agents, and other proven therapies that each reduce cardiovascular risk (Bhatt et al., 2019). In addition to these, demonstrating additional benefits for an NLRP3 inhibitor requires significantly larger trials than placebo-controlled studies. CANTOS enrolled over 10,000 patients for a median 3.7-year follow-up and demonstrated a 15% relative risk reduction with canakinumab, an investigation that exceeded most pharmaceutical company resources for an unproven mechanism. The question of whether pharmaceutical companies will invest heavily in large heart disease trials for NLRP3 drugs or focus instead on rare diseases is important for patients.

## 3 Natural product inhibitors and their mechanistic classification potential

### 3.1 Tier 1 confirmed direct inhibitors

Only a few natural products reported to affect NLRP3 have strong biochemical evidence supporting direct inhibition. These compounds deserve priority in development due to their established mechanisms, which facilitate rational structure-activity studies.

To date, oridonin, an ent-kaurane diterpenoid isolated from *Rabdosia rubescens* (Donglingcao), is the best-validated natural NLRP3 inhibitor. Its inhibitory mechanism is covalent, as mass spectrometry demonstrates that oridonin binds Cys279 in the NACHT domain, and mutation of this residue to alanine abolishes inhibition without affecting baseline inflammasome function (He et al., 2018). This covalent mechanism is distinct from that of non-covalent inhibitors, such as MCC950, and may provide prolonged target engagement after the compound is cleared. Functionally, it inhibits the NLRP3-NEK7 interaction, prevents oligomerization, and reduces the release of IL-1β and IL-18 in various cells. *In vivo*, oridonin (3–20 mg/kg) reduces inflammation in peritonitis, gout, and type 2 diabetes models (He et al., 2018) and attenuates myocardial fibrosis and cardiac remodeling in a murine MI model (Gao et al., 2021). Although oridonin has a distinct mechanism, it has significant pharmaceutical limitations that must be overcome for clinical use. It has very low aqueous solubility (<1 μg/mL), and current formulations only marginally improve its bioavailability. Due to limited oral bioavailability in rodents (<5%), most do not reach the circulation. It is rapidly metabolized in the liver, producing metabolites with unclear activity. These limitations require significant structural optimization or advanced delivery strategies before clinical translation.

Tranilast, a tryptophan metabolite analog marketed in Japan since the 1970s, inhibits NLRP3 through a distinct mechanism. This mechanism involves disrupting the NLRP3-NEK7 protein-protein interaction, as validated by crystallographic studies (Huang et al., 2018). This mechanism for inhibiting protein-protein interactions is fundamentally different from ATP-competitive approaches, potentially offering advantages in terms of resistance development and target specificity. Despite its modest potency, it is a viable candidate for accelerated development owing to its decades of clinical safety data and cardiovascular experience in coronary stent restenosis trials.

### 3.2 Tier 2 probable direct inhibitors

Numerous natural products have been demonstrated to inhibit cellular NLRP3, supported by computational docking or indirect binding evidence. However, they lack the definitive biochemical validation required for Tier 1. These compounds are promising candidates for further mechanistic studies to confirm or exclude direct NLRP3 binding.

Celastrol, a quinone methide triterpene from *Tripterygium wilfordi*i (Thunder God Vine), has attracted significant attention owing to its potent anti-inflammatory effects in various disease models. Molecular docking simulations indicate favorable NLRP3 NACHT domain binding with predicted binding energies comparable to those of validated inhibitors (Cascão et al., 2017). However, celastrol also inhibits heat shock protein 90 (HSP90), including by directly regulating the HSP90-NLRP3 interaction (Yang et al., 2025), the proteasome, and NF-κB (Lu et al., 2021; Yu et al., 2017), making it challenging to attribute its effects to NLRP3 specifically. However, this extensive multi-target profile, while previously considered beneficial therapeutic polypharmacology in some contexts, is a severe limitation for targeted cardiovascular therapy. Like indirect synthetic inhibitors, the lack of absolute target selectivity limits their clinical translation due to unpredictable safety profiles. Significant cytotoxicity further narrows the therapeutic window.

Parthenolide, a sesquiterpene lactone derived from *Tanacetum parthenium*, contains an electrophilic α-methylene-γ-lactone moiety that forms covalent protein cysteine adducts. This reactivity implies that it may covalently inhibit NLRP3, similar to oridonin, and competitive binding data provide preliminary support for direct NLRP3 binding (Juliana et al., 2010). However, its electrophilicity arises from reactions with glutathione and other cellular thiols, which may explain its known NF-κB inhibition via IKKβ modification. This lack of selectivity challenges whether it can specifically target NLRP3 among the thousands of cysteine residues in the proteome.

Sulforaphane, an isothiocyanate abundantly present in cruciferous vegetables, demonstrates NLRP3 inhibitory activity in numerous experiments and has entered clinical trials for inflammatory conditions (Zhou et al., 2024). Its anti-inflammatory properties are primarily associated with nuclear factor erythroid 2-related factor 2 (Nfr2) activation and upregulation of antioxidant genes. The Nrf2-NLRP3 regulatory axis is widely recognized as a key interface in modulating the inflammasome (Tastan et al., 2022). However, recent research suggests that it may also directly inhibit NLRP3, independent of Nrf2 (Greaney et al., 2016). Whether sulforaphane directly inhibits NLRP3 or acts indirectly through antioxidant effects remains unclear; however, its established safety profile as a dietary supplement supports clinical evaluation even without definitive mechanistic understanding.

### 3.3 Tier 3 functional inhibitors lacking direct binding evidence

This category includes natural products that act through indirect mechanisms, such as reducing NLRP3 activation in cellular assays without any direct binding.

Berberine, an isoquinoline alkaloid from *Coptis chinensis* and *Berberis* species, is anticipated to be a promising NLRP3-NEK7 interaction modulator because of its binding to NEK7, an essential co-factor for NLRP3 activation (Zeng et al., 2021). This interaction disrupts the NLRP3-NEK7 interface, thereby establishing a protein-protein interaction (PPI) blocking mechanism that is distinct from ATP-pocket binders. In experimental models, berberine reduces NLRP3-induced inflammation associated with cardiovascular and metabolic diseases. It reduces cardiac inflammation and fibrosis in AF and acute kidney injury (Yang et al., 2024) and suppresses NLRP3 activation in diabetic nephropathy and liver injury (Ali and Datusalia, 2024). However, berberine indirectly affects inflammasome priming by regulating other pathways, including 5′AMP-activated protein kinase (AMPK) and Nrf2 (Zhou et al., 2017; Dinesh and Rasool, 2017). This broad-spectrum activity presents a challenge in determining whether its *in vivo* effects are solely due to specific NLRP3-NEK7 interface disruption, a challenge shared with other Tier 3 candidates, such as curcumin.

Quercetin and related dietary flavonoids are among the most extensively studied natural products that modulate the inflammasome. These compounds exhibit potent antioxidant activity that can suppress reactive oxygen species (ROS)-dependent NLRP3 activation without direct target binding; however, significant effects on NF-κB, MAPK, and PI3K/Akt signaling pathways complicate mechanistic attribution (Wang et al., 2025; Wu et al., 2024). Molecular docking predicts favorable NLRP3 binding, but the interpretive value of computational predictions is limited by the promiscuous flavonoid binding to diverse protein targets. The failure of antioxidant strategies in cardiovascular outcome trials warrants caution regarding compounds that exert their effects through ROS scavenging rather than direct NLRP3 binding.

Curcumin has been proposed as an NLRP3 inhibitor, as it reduces IL-1β release in experimental systems (Ding et al., 2018). However, its poor bioavailability, rapid metabolism, and chemical instability raise questions about whether these effects result from target engagement or from confounding factors (Nelson et al., 2017). Curcumin is considered a pan-assay interference compound (PAINS) because of its promiscuous activity in screens, fluorescence that interferes with assay readouts, and aggregation at concentrations frequently used in research (Kaur et al., 2024). These issues do not preclude NLRP3 inhibition; however, they require experimental rigor that has often been absent in published studies.

Resveratrol, a polyphenol found in grapes and red wine, reduces NLRP3 activation in acute lung injury, myocardial injury, cerebral ischemia, and gout models (Fan et al., 2021; Maayah et al., 2021). Its effects are mediated by an indirect mechanism, including SIRT1 activation, mitophagy induction, and Nrf2 pathway upregulation, which suppresses inflammasome signaling without direct NLRP3 interaction (Cai et al., 2025). The direct binding of resveratrol with NLRP3 is not supported by any biochemical or structural evidence. Like many polyphenols, it has multiple known mechanisms, including AMPK activation, NF-κB suppression, and modulation of mitochondrial function, which could potentially inhibit NLRP3 without direct binding.

### 3.4 Structure-activity relationships and optimization strategies

Rational optimization may be facilitated by the recurrence of numerous structural features in natural product NLRP3 inhibitors. Electrophilic centers that covalently modify the cysteine, including the α,β-unsaturated carbonyl group in oridonin and the α-methylene-γ-lactone group in parthenolide, provide sustained target engagement. Covalent inhibitors have demonstrated success in other areas (for instance, EGFR kinase inhibitors and caspase-1 blockers), suggesting that electrophilicity can be adjusted to improve selective NLRP3 targeting with minimal off-target reactivity. The advantages of covalent binding with improved selectivity can be achieved through medicinal chemistry strategies that use controlled-reactivity warheads, such as acrylamides and chloroacetamides.

The pharmaceutical properties of natural scaffolds can be improved through semi-synthetic modification without compromising target engagement. Water-soluble oridonin prodrugs improved bioavailability in early studies; however, whether they retain Cys279-specific binding remains to be validated. Similar efforts to reduce the cytotoxicity of celastrol via functional group modification have demonstrated inconsistent retention of anti-inflammatory potency. Optimizing the drug-like properties of natural products while retaining the activity-determining structural features is a standard challenge in medicinal chemistry.

**FIGURE 2:**
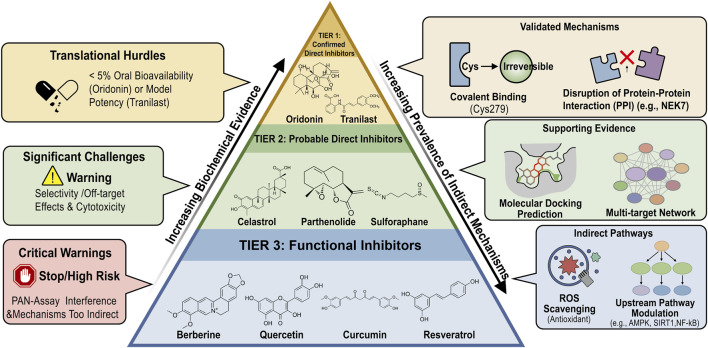
Mechanistic classification of natural product NLRP3 modulators. The three-tier system categorizes candidates based on the rigor of biochemical and structural validation. Tier 1 (Confirmed Direct Inhibitors) includes compounds with definitive evidence of direct NLRP3 engagement, such as oridonin, which facilitates irreversible covalent modification at Cys279, and tranilast, which disrupts the NLRP3-NEK7 protein-protein interaction (PPI). Pharmacokinetic limitations, particularly low oral bioavailability, remain the primary translational hurdle for these established binders. Tier 2 (Probable Direct Inhibitors), such as celastrol and parthenolide, exhibit favorable binding characteristics in computational or functional models but lack conclusive biophysical confirmation. These scaffolds frequently encounter challenges regarding target selectivity and potential cytotoxicity. Tier 3 (Functional Inhibitors) encompasses widely studied phytochemicals like quercetin and curcumin that suppress inflammasome activation via indirect pathways (e.g., ROS scavenging or upstream signaling) without direct target binding (Original figure created with Microsoft PowerPoint).

The chemical structures and proposed binding modes of representative natural product NLRP3 inhibitors are illustrated in Figure 2, and a complete mechanistic classification of natural product candidates is provided in Table 2.

**TABLE 2:** Natural product NLRP3 inhibitors: Mechanistic classification.

Tier 1: Confirmed direct inhibitors					
Compound	Plant source	Binding evidence	IC50	CV preclinical data	Challenges
Oridonin	*Rabdosia rubescens*	Covalent (Cys279); MS confirmed	∼0.5 μM	Atherosclerosis ↓50%; MI protection	Bioavailability <5%
Tranilast	*Synthetic analog*	Co-crystallography; SPR	20–50 μM	Stent restenosis (clinical)	Modest potency

Tier 2: Probable direct inhibitors					
Compound	Plant source	Supporting evidence	IC50		Selectivity concerns
Celastrol	*Tripterygium wilfordii*	Molecular docking; thermal shift	0.1–1 μM		HSP90, proteasome NF-κb
Parthenolide	*Tanacetum parthenium*	Electrophilic warhead	1–5 μM		IKKβ, multiple thiols
Sulforaphane	*Cruciferous vegetables*	Nrf2-independent component	1–10 μM		Nrf2 confounding

Tier 3: Functional inhibitors (no direct binding evidence)					
Compound	Plant source	Likely mechanism			Recommendation
Berberine	*Coptis chinensis*	AMPK/NF-κb suppression; NEK7-mediated PPI modulation			Requires binding studies
Quercetin	Multiple dietary	Antioxidant; NF-κB/MAPK			Not recommended
Curcumin	*Curcuma longa*	Multiple indirect			PAINS compound
Resveratrol	*Vitis vinifera*	SIRT1; antioxidant			Mechanism too indirect

## 4 Cardiovascular applications: bridging the gap from laboratory to clinic

### 4.1 Atherosclerosis

Atherosclerosis has the strongest scientific rationale for NLRP3 inhibition but is also the most difficult to translate to the clinic, a paradox that shapes development strategies across the field.

The argument for targeting NLRP3 in atherosclerosis is based on an unusually complete evidence chain from molecules to humans. Cholesterol crystals directly activate NLRP3, connecting lipid accumulation to inflammasome biology. The spatial localization of NLRP3 expression to regions of active inflammation and impending rupture is evident in human pathological specimens, which demonstrate higher levels in vulnerable plaques than in stable lesions (Paramel Varghese et al., 2016). Recent clinical data from 232 patients further demonstrate that serum NLRP3 and IL-1β levels independently predict unstable plaques (including rupture and erosion) and subsequent major adverse cardiovascular events (MACE) during a 14-month follow-up. The area under the curve (AUC) values are 0.874 for NLRP3 and 0.870 for IL-1β (Liu et al., 2026). Plaque burden and inflammation are consistently reduced in preclinical studies that use NLRP3 knockout or MCC950, thereby confirming a causal role in disease progression. With advancements in delivery technologies, biologic therapies are exploring direct intracellular targeting. For example, a novel bispecific antibody targeting both IL-1R1 and intracellular NLRP3 (InflamAb) has recently demonstrated efficacy in ApoE-deficient mice. This antibody reduces plaque development and enhances plaque stability parameters without the hepatotoxicity concerns associated with MCC950 (Delfos et al., 2025). Notably, the CANTOS trial demonstrated that neutralization of IL-1β, the principal downstream effector of NLRP3 activation, reduced recurrent cardiovascular events in patients with a prior MI, providing clinical proof that therapeutic anti-inflammatory intervention can significantly affect hard cardiovascular endpoints (Ridker et al., 2017). However, it is imperative not to conflate the success of a downstream biological antibody with the guaranteed success of upstream small-molecule direct inhibitors. To date, human outcome data confirming that direct NLRP3 inhibition safely reduces MACE are entirely absent.

Despite this robust scientific foundation, no Phase III clinical trial has been registered to assess the direct NLRP3 inhibitors in atherosclerotic cardiovascular disease. This indicates the practical challenges that have deterred pharmaceutical investment. MACE trials require monitoring of thousands of patients for years. CANTOS enrolled 10,000 patients and followed them for a median of 3.7 years, incurring a cost of more than one billion dollars. This scale surpasses the resources and timelines of most companies (Ridker, 2018). The additional benefits of the new drug are limited due to the availability of effective therapies for patients, and even larger trials are required to identify these benefits. Therefore, the problem is further exacerbated because of the lack of validated surrogate endpoints, which excludes smaller proof-of-concept studies.

### 4.2 AMI

AMI is a key target for NLRP3 inhibition. DAMPs, including ROS and extracellular ATP, activate NLRP3 in cardiomyocytes and infiltrating immune cells during reperfusion. This process initiates an inflammatory cascade that extends injury beyond ischemia alone (Liu et al., 2014). Stimulator of interferon genes (STING) has been identified as an upstream regulator of NLRP3-mediated pyroptosis in this context (Zhang et al., 2026).

Multiple animal models have demonstrated that NLRP3 inhibition at the reperfusion time reduces infarct size and preserves cardiac function. A selective NLRP3 inhibitor (16673-34-0) administered during reperfusion reduced infarct size by 56% in mice (Toldo et al., 2016). Similar protection was conferred by genetic deletion of NLRP3 or its downstream effector caspase-1 (Kawaguchi et al., 2011). The selective inhibitor MCC950 reduced infarct size by approximately 50% and preserved ejection fraction in a porcine model, thereby providing large-animal validation (van Hout et al., 2017). MCC950 also suppresses microvascular thromboinflammation, regardless of reflow status. Notably, recent studies indicate that there may be sex-specific differences in response, with estrogen-mediated NLRP3 inhibition leading to cardioprotection in female models (Cheng et al., 2025).

Clinical translation faces timing challenges. Animal studies require inhibitor administration at or before reperfusion due to a narrow therapeutic window. However, percutaneous coronary intervention is a standard therapy for patients with STEM performed within 60–120 min of their hospital arrival. This time is insufficient for oral medication to achieve therapeutic levels. This could be resolved through intravenous formulations; however, most development programs primarily focused on oral dosing. Despite these challenges, clinical proof-of-concept is emerging. A phase II trial of selnoflast, an oral NLRP3 inhibitor, in post-MI patients with residual inflammation (hsCRP ≥2 mg/L)demonstrated a 36% reduction in IL-6 and a 70% reduction in hsCRP without any safety concerns (Kunder et al., 2025). While these surrogate biomarker reductions are mechanistically encouraging, they do not inherently guarantee clinical benefit. The cardiovascular field has historically seen agents that lower inflammatory markers fail to improve MACE, underscoring the need for rigorous outcome-driven trials for selnoflast. A meta-analysis of 32 trials (37,056 patients) confirmed that anti-inflammatory therapies targeting the NLRP3/IL-1β/IL-6 pathway reduce recurrent MI (RR 0.85) and revascularization (RR 0.80) (Pan et al., 2025). In a STEMI pilot study, colchicine reduced infarct size through indirect inhibition of NLRP3 by suppressing ASC polymerization (Deftereos et al., 2015). It is now a recommended guideline for secondary prevention (Nidorf et al., 2020). Numerous novel NLRP3 inhibitors are currently in clinical development.

### 4.3 Heart failure

Heart failure is characterized by persistent low-grade inflammation, which drives progressive myocardial remodeling and functional decline. This makes it a logical target for anti-inflammatory therapy; however, long-term therapies raise safety issues that current data cannot completely address.

Inflammatory mechanisms in heart failure have been well-documented. Patients exhibited elevated levels of circulating cytokines, including IL-1β, IL-6, and TNF-α, which are associated with disease severity and worse outcomes (Dick and Epelman, 2016). Numerous studies have demonstrated that genetic variants in NLRP3 pathway genes are associated with heart failure risk, implicating NLRP3 inflammasome activation in heart failure pathogenesis.

Although clinical data on NLRP3 inhibition in heart failure are limited, they are now emerging. A Phase Ib trial of the oral NLRP3 inhibitor dapansutrile in patients with stable systolic heart failure (NYHA II-III, left ventricular ejection fraction (LVEF) ≤ 40%) demonstrated good safety and tolerability over 14 days. In the 2000 mg cohort, LVEF improved from 31.5% to 36.5%, and exercise time increased from 570 to 616 s (Wohlford et al., 2020). The trial is registered under the number NCT03534297. (ClinicalTrials.gov. Study of Dapansutrile Capsules in Heart Failure. NCT03534297. Available from: https://clinicaltrials.gov/ct2/show/NCT03534297). However, it must be strongly emphasized that this was a short-term (14-day) safety study with a small sample size. These functional improvements are exploratory and hypothesis-generating; adequately powered, long-term Phase II/III trials are strictly required before any definitive conclusions regarding its efficacy in reversing heart failure remodeling can be drawn.

The safety considerations for chronic NLRP3 inhibition differ from those for short-term use in acute settings. Heart failure therapies are prescribed for years or decades, with the potential for cumulative toxicity and drug interactions with standard treatments, including statins, beta-blockers, RAAS inhibitors, and SGLT2 inhibitors. In the CANTOS trial, IL-1β blockade in other populations suggests that canakinumab increased fatal infections (Ridker et al., 2017). It is uncertain whether direct NLRP3 inhibitors will offer a better safety profile than IL-1β blockade and whether they will require long-term data specific to heart failure populations.

### 4.4 Emerging cardiovascular indications

Recurrent pericarditis, characterized by episodic chest pain and pericardial inflammation that frequently recurs despite standard therapy, is a particularly promising near-term application. The initiation of the 2025 VTX2735 Phase II trial reflects the recognition that this condition offers development advantages, including clear symptomatic endpoints, a relatively short time to event occurrence, and limited effective treatment options (Ventyx Biosciences. VTX2735 Phase II trial initiation for recurrent pericarditis. Press release, January 2025).

Diabetic cardiomyopathy is another underexplored area. NLRP3 activation is induced by various conditions, including hyperglycemia, advanced glycation end products, and ectopic cardiac lipid deposition, which adversely affect diabetic patients by contributing to diastolic dysfunction and HFpEF (Wang et al., 2024). AF, in which inflammatory signaling promotes atrial remodeling and arrhythmogenesis, similarly represents an opportunity. Colchicine efficacy in reducing postoperative AF suggests that more selective inflammasome targeting could produce superior results.

## 5 Challenges, opportunities, and future directions

### 5.1 Pharmacokinetic optimization

Poor pharmacokinetics affect most current NLRP3 inhibitors, whether they are synthetic or natural. This is a common problem that all successful candidates must resolve. The root cause lies in the physical properties of the NLRP3-binding pocket, which is predominantly hydrophobic as indicated by the structural studies. Compounds that bind well tend to be lipophilic, with LogP value >3, resulting in poor aqueous solubility, limiting oral absorption, and making formulation development challenging. The problem is compounded as NLRP3 is intracellular; compounds must dissolve in biological fluids and cross cell membranes to achieve effective cytosolic concentrations (Tengesdal et al., 2024). Natural products are particularly problematic: oridonin has an oral bioavailability of <5%, and celastrol has approximately 10%.

Advanced delivery systems may prove advantageous. Nanoparticle formulations (liposomes, PLGA nanoparticles, and solid lipid nanocarriers) can improve bioavailability and alter tissue distribution for poorly soluble compounds (Ramalingam, 2023; Mitchell et al., 2021). Targeted delivery strategies using ligands for macrophage receptors (for instance, mannose and folate) could deliver drugs to sites of inflammasome activation, thereby reducing systemic exposure and toxicity.

### 5.2 Selectivity and off-target effects

The relationship between selectivity and safety for NLRP3 inhibitors is not simple. MCC950 demonstrated that high *in vitro* selectivity does not guarantee *in vivo* tolerability. MCC950 is instructive as it exhibited IC50 ratios over 1000-fold against other inflammasomes and no activity in broad pharmacological screens. However, Phase II trials revealed dose-limiting hepatotoxicity that was not predicted by preclinical studies (Zheng et al., 2024). Subsequent chemical proteomics demonstrated that MCC950 binds CA2 at clinically relevant concentrations, which may provide an explanation. Notably, this revealed that conventional selectivity analysis failed to identify various safety-related protein interactions. Natural products face different selectivity problems. While plant secondary metabolites have evolved to interact with multiple targets, this broad-spectrum activity must not be misconstrued as an advantage. For direct cardiovascular therapeutics, absolute target specificity is paramount to preserve basic immune defenses, and the promiscuity of many natural products remains a major translational bottleneck. Plant secondary metabolites have evolved to interact with multiple targets in different organisms, rather than to target one human protein (Cascão et al., 2017). Depending on the context, this broad-spectrum activity may be perceived as therapeutic polypharmacology or as problematic promiscuity. The specific disease determines whether it is beneficial or detrimental.

### 5.3 Integration of artificial intelligence and systems biology

The discovery of NLRP3 inhibitors is being revolutionized by computational methods, particularly in the context of natural products. Machine learning models for virtual screening have improved. Random forest models trained on NLRP3 inhibitor datasets have identified new active compounds when screened against natural product libraries and have achieved AUC >0.83 in cross-validation (Shi et al., 2023; Alzarea, 2025). Molecular structures can be directly processed by deep learning models using graph neural networks, without requiring pre-defined descriptors. NLRP3 is analyzed within the context of its broader signaling network through systems biology approaches. Network pharmacology integrates compound-target binding data with protein interaction networks and pathway databases to predict the effects of multi-target drugs (Liao et al., 2025). Single-cell RNA sequencing of atherosclerotic plaques and failing hearts has identified the cell types that express NLRP3 and related genes, implying potential for cell-specific delivery.

## 6 Conclusion and perspectives

The NLRP3 inflammasome has emerged as a critical mediator of cardiovascular pathology; however, translating direct inhibitors from preclinical promise to clinical application has been more challenging than initially anticipated. Despite the exceptional *in vitro* selectivity of MCC950, its failure due to hepatotoxicity underscores a fundamental limitation of current preclinical models. It highlights the need for early off-target profiling using chemical proteomics before initiating costly trials. This experience enables the evaluation of natural product inhibitors, in which our three-tier evidence classification reveals a significant gap between compounds that have been confirmed to bind biochemically and those that exert their effects through indirect mechanisms. The stringent criteria for direct inhibition are met by only a few natural products, such as oridonin, which covalently modifies Cys279, whereas widely studied flavonoids and polyphenols potentially act through antioxidant or multi-kinase pathways that complicate mechanistic attribution and limit their use as leads for structure-based optimization.

**FIGURE 3:**
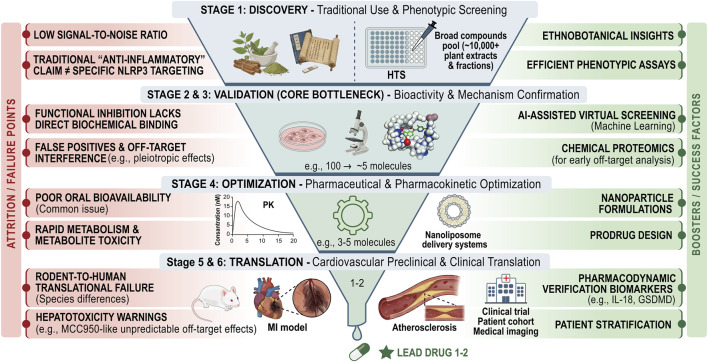
The translational pipeline for natural product-derived NLRP3 inhibitors in cardiovascular medicine. The progression of candidates from medicinal plants to clinical applications involves a stringent selection process across multiple stages. Discovery and Mechanism Validation (Stages 1–3) prioritize the identification of direct biochemical engagement to distinguish true inhibitors from functional modulators acting through indirect mechanisms like ROS scavenging. Integration of machine learning and chemical proteomics serves to accelerate this validation. Pharmaceutical Optimization (Stage 4) addresses the inherent pharmacokinetic barriers of natural scaffolds, such as the low bioavailability and rapid metabolism observed with oridonin. Advanced nanoparticle formulations and prodrug strategies are utilized to enhance drug-like properties. Cardiovascular Translation (Stages 5–6) advances candidates from preclinical models of atherosclerosis and myocardial injury toward human clinical trials. The clinical failure of MCC950 highlights the necessity of evaluating species-specific off-target risks during translation. Future success depends on the implementation of validated NLRP3-specific biomarkers and stratified trial designs (Original figure created with BioRender.com).

Cardiovascular applications of NLRP3 inhibitors require indication-specific development strategies. AMI provides a defined therapeutic window, but it demands intravenous formulations that are not prioritized by most programs. Heart failure addresses a significant unmet need, but it requires long-term safety data that will take years to accumulate. Atherosclerosis has the strongest mechanistic rationale, but it necessitates prohibitively large outcome trials. The recent entry of VTX2735 into Phase II trials for recurrent pericarditis demonstrates a pragmatic approach to advancing, selecting indications with clear endpoints and shorter trial durations to establish clinical proof of concept before expanding to larger population cohorts. For natural product inhibitors, progress will depend on balancing mechanistic rigor with pharmaceutical optimization (Figure 3). For instance, covalent binding of oridonin provides an ideal template for structure-activity studies, but its <5% oral bioavailability demands the use of medicinal chemistry or advanced formulation strategies. Although the decades of clinical safety data of tranilast offer a unique advantage, its modest potency still leaves room for improvement. Ultimately, successful translation will require robust pharmacodynamic biomarkers, formulations that overcome the limitation of the hydrophobic NACHT pocket, and adaptive trial designs that facilitate efficient evaluation across multiple cardiovascular indications. Most importantly, while early clinical and preclinical data are mechanistically compelling, expectations regarding their cardiovascular translation must remain cautious. Definitive proof of their therapeutic value relies absolutely on future large-scale, long-term Phase III trials demonstrating unambiguous reductions in hard cardiovascular endpoints.
